# Vanadium Induces Oxidative Stress and Mitochondrial Quality Control Disorder in the Heart of Ducks

**DOI:** 10.3389/fvets.2021.756534

**Published:** 2021-10-26

**Authors:** Zhiwei Xiong, Chenghong Xing, Tianfang Xu, Yan Yang, Guohui Liu, Guoliang Hu, Huabin Cao, Caiying Zhang, Xiaoquan Guo, Fan Yang

**Affiliations:** ^1^Jiangxi Provincial Key Laboratory for Animal Health, Institute of Animal Population Health, College of Animal Science and Technology, Jiangxi Agricultural University, Nanchang, China; ^2^Jiangxi Agricultural Technology Extension Center, Nanchang, China; ^3^Ganzhou Agriculture and Rural Affairs, Ganzhou, China

**Keywords:** vanadium, oxidative stress, mitochondrial quality control, heart, duck

## Abstract

Vanadium (V) is an ultra-trace element presenting in humans and animals, but excessive V can cause toxic effects. Mitochondrial quality control (MQC) is an essential process for maintaining mitochondrial functions, but the relationship between V toxicity and MQC is unclear. To investigate the effects of excessive V on oxidative stress and MQC in duck hearts, 72 ducks were randomly divided into two groups, including the control group and the V group (30 mg of V/kg dry matter). The cardiac tissues were collected for the histomorphology observation and oxidative stress status evaluation at 22 and 44 days. In addition, the mRNA and protein levels of MQC-related factors were also analyzed. The results showed that excessive V could trigger vacuolar degeneration, granular degeneration, as well as mitochondrial vacuolization and swelling in myocardial cells. In addition, CAT activity was elevated in two time points, while T-SOD activity was increased in 22 days but decreased in 44 days after V treatment. Meanwhile, excessive V intake could also increase the number of Drp1 puncta, the mRNA levels of mitochondrial fission–related factors (Drp1and MFF), and protein (MFF) level, but decrease the number of Parkin puncta and the mitochondrial biogenesis (PGC-1α, NRF-1, and TFAM), mitochondrial fusion (OPA1, Mfn1, and Mfn2), and mitophagy (Parkin, PINK1, P62, and LC3B) related mRNA levels and protein (PGC-1α, Mfn1, Mfn2, PINK1) levels. Collectively, our results suggested that excessive V could induce oxidative stress and MQC disorder in the heart of ducks.

## Introduction

Vanadium (V) is an ultra-trace element that can affect the activities of intracellular enzymes and acts a crucial role in metabolism and normal cell function and development (1). Moreover, most studies indicated that the concentration of V below 1 × 10^−5^ M could be used as a potential therapeutic agent for the treatments of cancer or diabetes (2–4). However, due to the extensive use of V in industry, excessive V pollutes the environmental ecosystems and is enriched in the humans and animals via the food chain, subsequently causing various tissue and organ injuries, such as heart, kidney, and lung. The heart, one of the target organs of V's toxicity, and excessive V entering the body could cause serious damage to it (5). Moreover, many studies demonstrated that excessive V could cause the injury of cardiac tissues via promoting the generation of reactive oxygen species (ROS) (6, 7), which in turn leads to mitochondrial dysfunction by complex signaling mechanisms.

Mitochondria are highly dynamic double-membraned organelles, especially abundant in cardiac tissue, which takes part in numerous cellular processes, such as ATP production, bioenergetics, metabolism, redox balance, and cell death (8). They also can produce ROS via transferring electrons in the mitochondrial respiratory chain to the oxygen atoms (9). Meanwhile, efficient antioxidant systems neutralize the extra ROS to resist oxidative stress and protect mitochondria (10, 11). Most of the studies suggested that V induced the generation of excess ROS and impaired antioxidant defense systems, eventually causing oxidative stress and mitochondrial dysfunction (12, 13). However, the maintenance of mitochondrial function depends on the normal operation of the mitochondrial quality control (MQC) system.

MQC, a self-functioning security mechanism, maintains the functional integrity of mitochondria, including mitochondrial biogenesis, mitochondrial fission, mitochondrial fusion, and mitophagy. Mitochondrial biogenesis coordinates energy homeostasis and cell growth by generating new mitochondria from existing mitochondria through crosstalk between nuclear and mitochondrial genomes (14). In addition, mitochondrial fission and fusion are the critical processes to maintain the health of mitochondria and cells for keeping the steady-state morphology of the mitochondrial network, which were related to the changes in metabolic states and diseases (15). In addition, mitophagy participates in a course of mitochondrial repairment, in the process of which damaged portions of mitochondria were removed from the healthy parts by division, subsequently degraded via a specific mode of autophagy (16). Based on the aforementioned, these specific mechanisms of MQC are effective ways to promote mitochondria renewal to maintain cell homeostasis. As shown in a recent study, cadmium could cause necroptosis and tissue damage by promoting mitochondrial fission leads to MQC disorder (17). Manganese has been verified to induce mitochondrial dysfunction by enhancing the activation of mitophagy (18). Therefore, we realized that MQC plays an important role in the toxic mechanism of heavy metals. A previous study has indicated that excessive V could induce mitochondrial dysfunction (19), but the specific mechanism should be further explored.

Emerging evidence suggested that the toxicity mechanism of V is mainly manifested in the damage of high levels of ROS to mitochondria, leading to oxidative stress and mitochondrial dysfunction (20). However, the role of MQC in the mechanism is unclear. Therefore, we explored the impacts of V exposure on oxidative stress and MQC in the heart of ducks in this study via histomorphometry observation, transmission electron microscope observation, immunofluorescence detection analysis, antioxidant-related indicator detection, and MQC-related mRNA and protein level detection further to enrich the theory of the V-induced cardiac toxicity mechanism.

## Materials and Methods

### Experiment Animals and Management

The study was conducted according to ethical guidelines and approved by the animal ethics committee of Jiangxi Agricultural University (Approval ID: JXAULL-2020-32). All ducks have received the standard rations according to the National Research Council (NRC), which referred to Ren et al. (21). Dietary composition and nutrient levels are shown in Table 1. Before the formal experiment, all ducks' health were assessed by the clinical examination. All ducks were reared under duck feeding conditions with a light/dark cycle for 12 h, a relative humidity of 65%, a relative temperature of 25°C, and had *ad libitum* feeding and drinking in 44 days.

**Table 1:** The component and nutritional levels of the basal diet.

**Item (%, unless noted)**	**Content**
Corn	47
Wheat bran	13
Rice bran	9
Soybean meal, 43%	9
Rapeseed meal	9
Cottonseed meal	6
Rapeseed oil	2.88
Calcium carbonate	0.96
Dicalcium phosphate, 2H_2_O	1.275
l-Lysine-HCl	0.37
d, l-Methionine	0.226
Threonine, 98.5%	0.044
Tryptophan, 98.5%	0.032
Sodium chloride	0.4
Choline chloride, 50%	0.2
Bentonite	0.913
Mineral premix^1^	0.4
Vitamin premix^2^	0.2
**Analyzed nutrient content**	
ME (kcal/kg, calculated)	2,914
CP (analyzed)	17.12
Calcium (analyzed)	0.94
Total phosphorus (analyzed)	0.84
Nonphytate phosphorus (calculated)	0.478

1 *Dietary supply per kilogram: copper, 8 mg; iron, 80 mg; zinc, 90 mg; manganese, 70 mg; selenium, 0.3 mg; iodine, 0.4 mg*.

2 *Dietary supply per kilogram: vitamin A, 15,000 IU; vitamin D_3_, 5,000 IU; vitamin K_3_, 5 mg; vitamin E, 80 mg; vitamin B_1_, 3 mg; vitamin B_2_, 9 mg; vitamin B_6_, 7 mg; vitamin B_12_, 0.04 mg; nicotine acid, 80 mg; pantothenic acid, 15 mg; biotin, 0.15 mg; folic acid, 2 mg; vitamin C, 200 mg; 25-hydroxycholecalciferol, 0.069 mg*.

### Determination of Lethal Dose 50 (LD_50_)

The ammonium metavanadate (NH_4_VO_3_) was used as the source of V. Fifty-four 1-day-old ducks were randomly divided into nine groups for the determination of LD_50_. Different doses of V, i.e., 10, 50, 100, 500, 1000, 1500, 2000, 2500, and 3000 mg/kg V dry matter (DM), were given to these groups, respectively. The dose-dependent effects of mortality were observed daily. Ultimately, 125.35 mg/kg V DM was assessed to be the LD_50_ of NH_4_VO_3_.

### Toxicity Trials

Seventy-two healthy 1-day-old Peking ducks were randomly assigned to two groups: the control group (basal diet) and the V group (30 mg/kg V DM), which followed the principle of the half male and half female. According to the LD_50_ of V in the ducks and the previous study (22), we chose a more conservative dose of 30 mg of V/kg DM. Cardiac tissues of the ducks were collected on 22 and 44 days.

### Sample Collection

On days 22 and 44, cardiac tissues were collected after ducks were anesthetized with an overdose of the intravenous injection of sodium pentobarbital anesthesia. Subsequently, the cardiac tissues were flushed with normal saline (0.9% NaCl; Gemeiyan, China) and removed the surface fluid with filter paper. Finally, the parts of the cardiac tissues were put into cryotubes and stored at −80°C, and the rest were applied to histomorphology observation and immunofluorescence detection analysis (23).

### Histomorphology Observation

The cardiac tissue specimens with a volume of 2 × 2 × 0.3 cm^3^ (*n* = 6) on 44 days were fixed with 4% paraformaldehyde at room temperature for 48 h, and the specific method was described in Zhang's publication (24). Afterward, the slides were stained with H&E and the pathological changes in the heart were evaluated under a light microscope (Olympus, Japan) (25).

### Transmission Electron Microscope Observation

The experimental procedures referred to the method of description in Shi et al.'s (26) paper. In brief, the samples were fixed in 2.5% glutaraldehyde and 0.1 M sodium phosphate mixed buffer (pH 7.2), respectively, and then post set with 1% osmium tetroxide in sodium phosphate buffer. Subsequently, the specimens were graded, dehydrated with 50% ethanol, and treated twice with propylene oxide. Finally, the specimens were embedded in araldite to slice and stain. The pathological changes of the myocardial cell were observed under microscope transmission electron microscopy Zeiss 900 microscope (Zeiss, Germany) (27).

### Oxidative Stress Indices Determination

The levels of total superoxide dismutase (T-SOD), catalase (CAT), malonaldehyde (MDA), and hydrogen peroxide (H_2_O_2_) were measured using commercial kits (Nanjing Jiancheng Bioengineering Institute, China) according to the manufacturer's instructions.

### Immunofluorescence Detection Analysis

Immunofluorescence staining observation was carried out by Zhang et al. (23) as reference. In short, the sections were incubated with antibodies against Drp1 (1:100; Wanleibio, China) or Parkin (1:400; Proteintech, USA) at 4°C overnight, and incubated the fluorescein (FITC) conjugated goat anti-rabbit or anti-mouse immunoglobulin (Ig)G (1:1,000, diluted) after washing off excess antibody with phosphate-buffered saline (PBS). Finally, the sections were washed by PBS and strained with 4′,6-diamidino-2-phenylindole (DAPI). The fluorescence intensities of Drp1 and Parkin were observed under the fluorescence microscope (Nikon Eclipse C1, Tokyo, Japan) (28).

### Real-Time Quantitative Polymerase Chain Reaction (RT-qPCR) Analysis

The primers and sequences used for RT-qPCR are shown in Table 2. According to the manufacturer's protocol, approximately total RNA of 40 μg (*n* = 6) was isolated from cardiac tissue by applying a Trizol reagent (Vazyme, China) and then using GeneQuant 1,300 spectrophotometer determined the RNA concentration. The RNA of 1,000 ng was reverse-transcribed into cDNA using the TransScript Uni All-in-One First-Strand cDNA Synthesis Super Mix (Trans, China) according to the manufacturer's instructions. Then RT-qPCR reaction was performed using 2× SYBR Green qPCR Master Mix (Servicebio, China) and carried out using QuantStudio7 Flex Real-Time PCR Systems (ABI 7900HT; Applied Biosystems, USA). The changes of the relative mRNA levels were assessed using the 2^−ΔΔCT^ method and standardized by GAPDH (29).

**Table 2:** Premier sequences used for real-time PCR.

**Gene**	**5^**′**^-Primer (F)**	**3^**′**^-Primer (R)**
PGC-1α	GTCTGCCTCTGCGCGAC	CCAGAGCAGCACACTCGAT
NRF-1	AGACGCGTTTGCTACGGAAG	TGTGCCTGGGTCATCTTGTC
TFAM	GGAGTTTCACCTGTGGCAAA	TCCACGTAGTGGAGCTTTGG
OPA1	GGATCTGCTGTAGGTGGTGG	TCACTAACCAAATTGTAACCAGGA
Mfn1	GCTGTGTACGAGGCAGCTAT	GTTCCTGTATGTTGCTTCCACG
Mfn2	GGAAGGGAGGAAAGCGCAATG	CCAGTACCTGCTCTTCTGTGG
Drp1	AAGTGGCCTGAGGTGGATGA	CGGGTTCTCCACAGTTTCACT
MFF	TCGACTAAGGATTGCACTGTGA	TGGTAAGCCCTACGAGTGGA
PINK1	GCTGTGTACGAGGCAGCTAT	CGAAGAACCAGCCGAGATGT
Parkin	CGCCGCCATGATAGTGTTTGTT	TTCTGCAGCGTCAAGTCGTT
P62	TCCCTTCTTGGTTCAACGGG	GAAGGGGCACCAAGTGAGAG
LC3B	ACAGTACAGACGAGCACCTC	CCAGAAAACTGTCACACGCA
GAPDH	TGATGCTCCCATGTTCGTGA	CTTTTCCCACAGCCTTAGCAG

*F, forward, R, reverse*.

### Western Blot Analysis

According to the manufacturer's protocol, the total protein was isolated from the 44-day cardiac tissue using RIPA lysis buffer (Solarbio, China), which contains protease inhibitors (PMSF) (Servicebio, China) (30). Subsequently, we measured the protein concentrations by using the bicinchoninic acid protein assay kit (Solarbio, China). Equal protein samples (10 μg) were boiled for 10 min after mixing with the 6× SDS-PAGE loading buffer (TransGen Biotech, China), and run on 12% SDS-polyacrylamide denaturing gels and transferred onto polyvinylidene fluoride (PVDF) membranes (Biosharp, China) (31). After blocking with tris buffered saline tween (TBST) containing 5% non-fat milk powder for 2 h at room temperature, the membrane was incubated overnight with diluted primary antibodies against PGC-1α (1:5,000; Proteintech, USA), Mfn1 (1:2,000; Proteintech, USA), Mfn2 (1:2,000; Wanleibio, China), Drp1 (1:1,500; Wanleibio, China), PINK1 (1:500; Bioss, China), and GAPDH (1:5,000; Proteintech, USA) at 4°C for 12 h. Subsequently, these membranes were washed by PBS adding tween-20 and incubated with corresponding second antibodies for 2 h. The bands were visualized by using enhanced chemiluminescence (ECL) reagents (Vazyme Biotech, USA). The signal was detected with Image Lab Software (Bio-Rad, USA), and protein levels were analyzed by ImageJ software. The quantitative analysis of each band was normalized in its respective loading control (GAPDH).

### Statistical Analysis

The data were expressed as the mean ± SD. All data were calculated by Microsoft Excel 2016 and homogenous data were analyzed by one-way ANOVA using SPSS 25.0 (SPSS Inc., Chicago, IL, USA), in which the significances of intergroup differences were analyzed via least-significant difference analysis. All bar graphs were drawn using the GraphPad Prism 8.0 (GraphPad Inc., La Jolla, CA, USA). The *p*-value < 0.05 was considered statistically significant unless otherwise stated.

## Results

### The Effects of V on Histopathology in the Heart of Ducks

**Figure 1:**
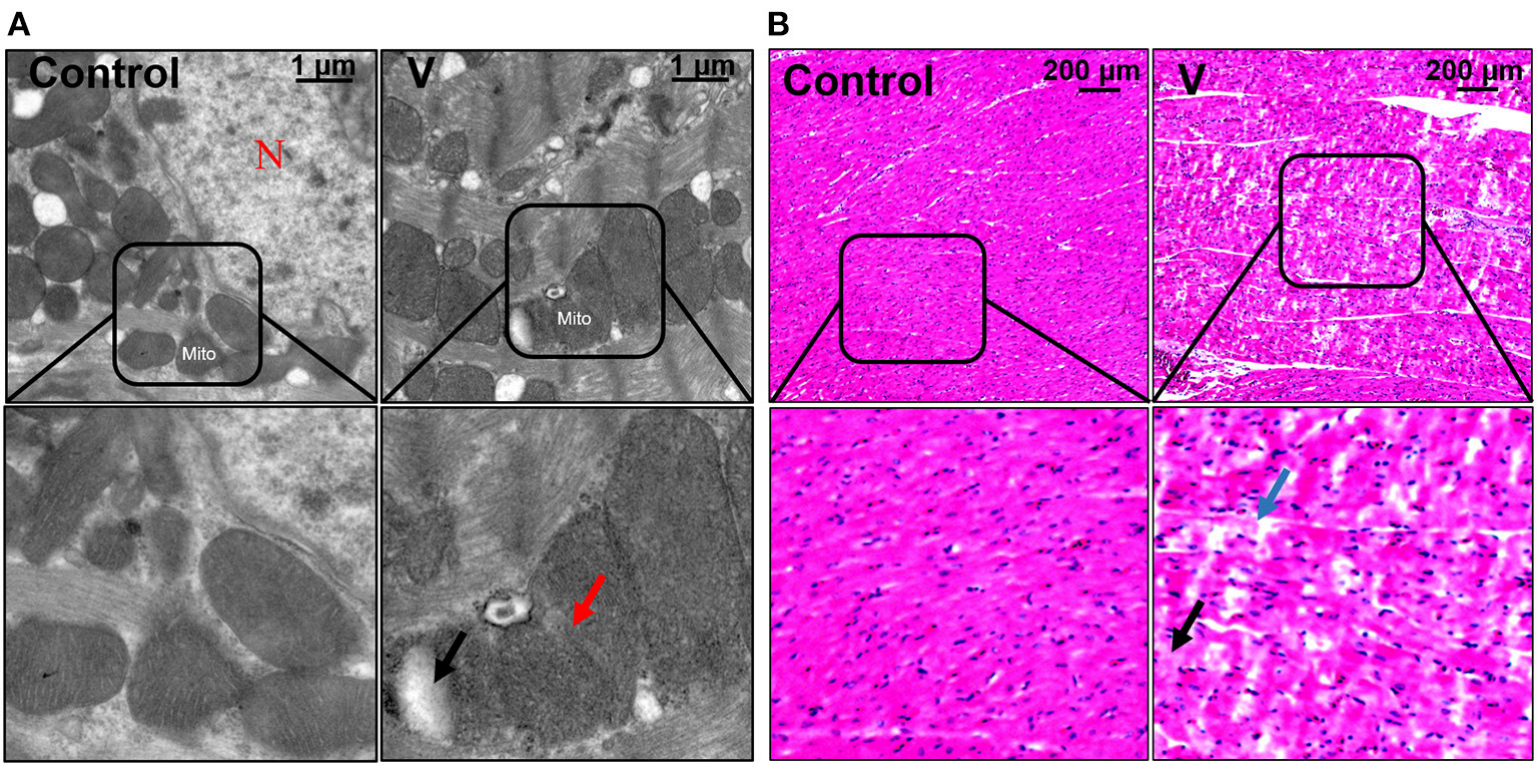
The changes of histomorphology in the heart of ducks on 44 days. **(A)** TEM observation. The scale bar is 1 μm. The red arrow refers to mitochondrial swelling. The black arrow refers to mitochondrial vacuolization. N, nucleus; Mito, mitochondria. **(B)** Pathological observation of the hearts in ducks. Scale bar, 200 μm. The black row indicates granular degeneration of cardiomyocytes. The blue row indicates the vacuolar degeneration of cardiomyocytes.

The ultrastructural and pathological changes of heart in ducks on 44 days are shown in Figure 1. According to the results of the histopathological examination in the heart, the cardiomyocytes were arranged neatly and orderly in the control group, but V exposure induced vacuolization and granular degeneration in the myocardial cells on 44 days (Figure 1B). In addition, the ultrastructural observation of the myocardial cells showed that the structure of organelles and nucleus were clear in the control group, and mitochondrial swelling and vacuolization were observed under ultrastructure in the V group on 44 days (Figure 1A).

### The Effects of V on Oxidative Stress Status in the Heart of Ducks

**Figure 2:**
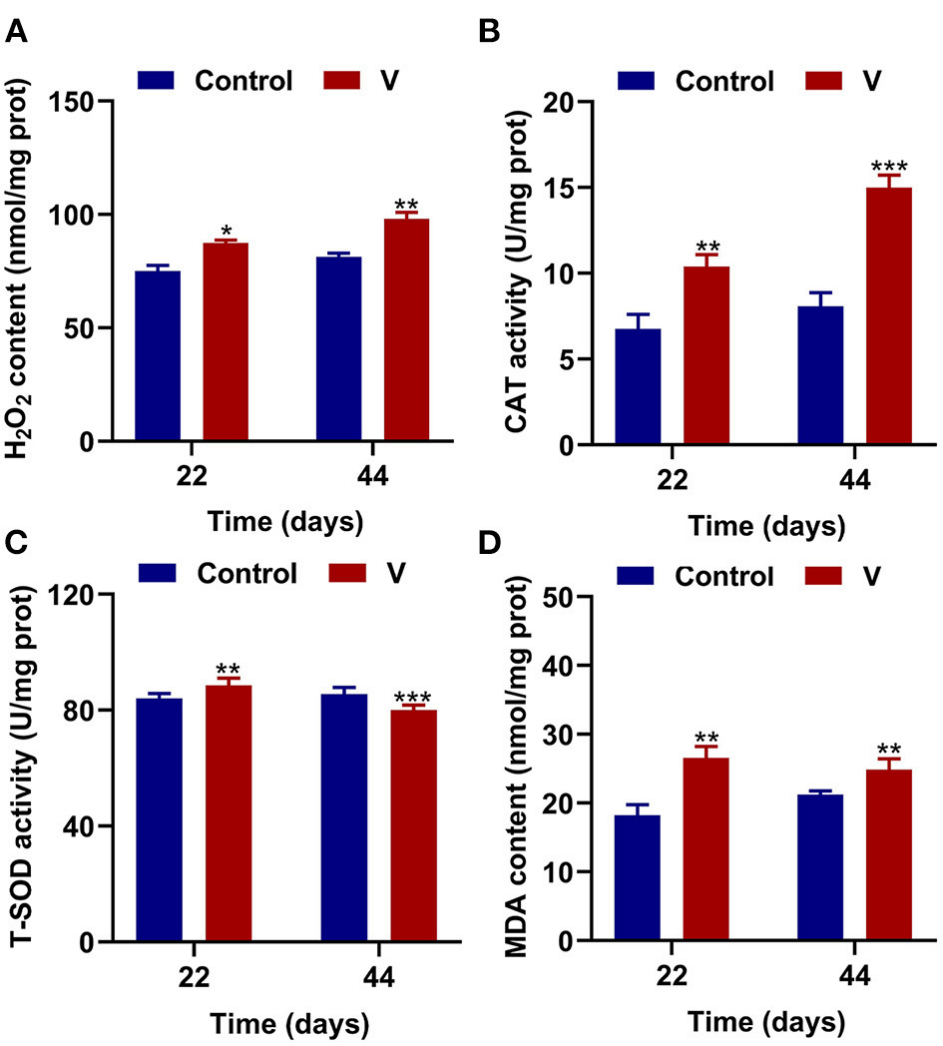
V induced oxidative stress in the heart of ducks. **(A)** H_2_O_2_ content. **(B)** The activity of CAT. **(C)** The activity of T-SOD. **(D)** MDA content. Data are represented as mean ± SD (*n* = 6 per group). “*” Indicates significant difference compared with the corresponding control (*p < 0.05, **p < 0.01, and ***p < 0.001).

The results showed that excessive V significantly (*p* < 0.05, *p* < 0.01, or *p* < 0.001) increased the levels of H_2_O_2_, MDA, and CAT on 22 and 44 days (Figures 2A,B,D). However, T-SOD activity was observably (*p* < 0.01 or *p* < 0.001) increased on 22 days but decreased on 44 days in the V group compared with the control group (Figure 2C).

### The Effects of V on Mitochondrial Biogenesis in the Heart of Ducks

**Figure 3:**
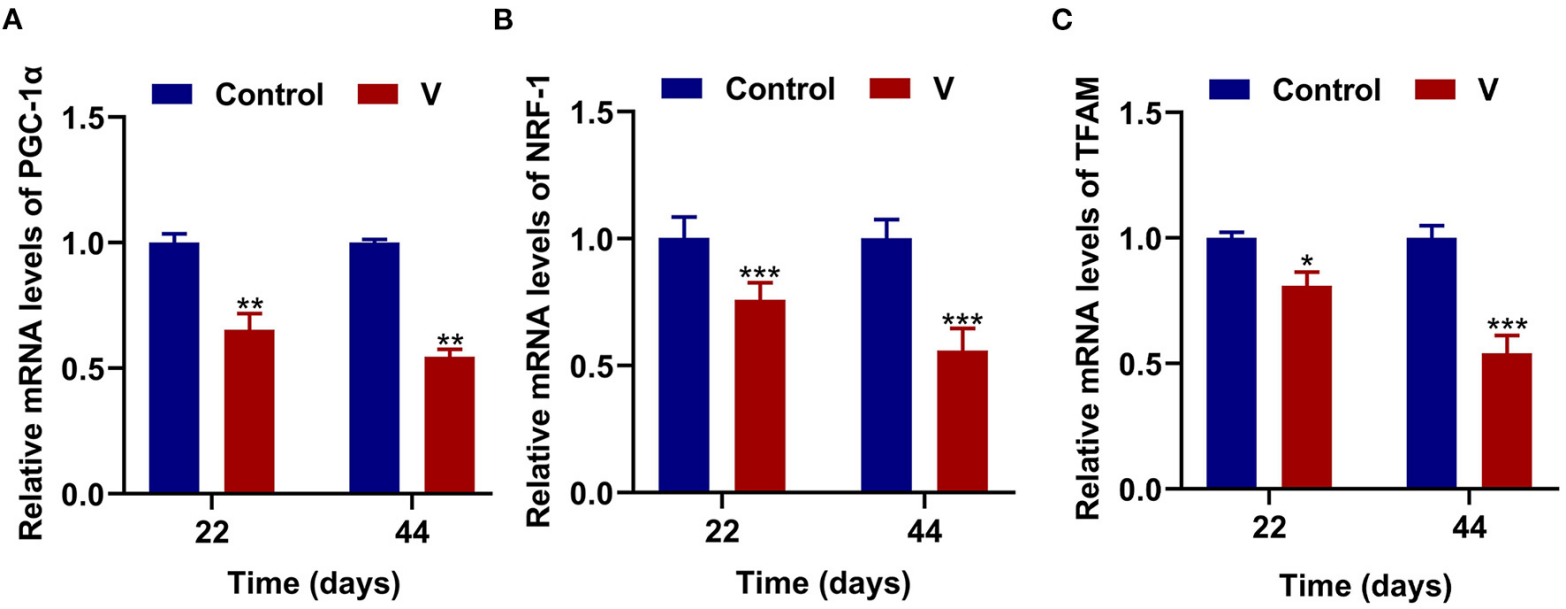
The effects of V on mitochondrial biogenesis in the heart of ducks. **(A)** The mRNA levels of PGC-1α. **(B)** The mRNA levels of NRF-1. **(C)** The mRNA levels of TFAM. *p < 0.05, **p < 0.01, and ***p < 0.001.

As shown in Figure 3, V exposure dramatically (*p* < 0.05, *p* < 0.01, or *p* < 0.001) decreased the mRNA levels of mitochondrial biogenesis (PGC-1α, NRF-1, and TFAM) on 22 and 44 days. Moreover, the PGC-1α protein level in the V group was also reduced significantly (*p* < 0.001) in comparison with the control group on 22 and 44 days (**Figures 6A,B**).

### The Effects of V on Mitochondrial Fission and Fusion Related Factors in the Heart of Ducks

**Figure 4:**
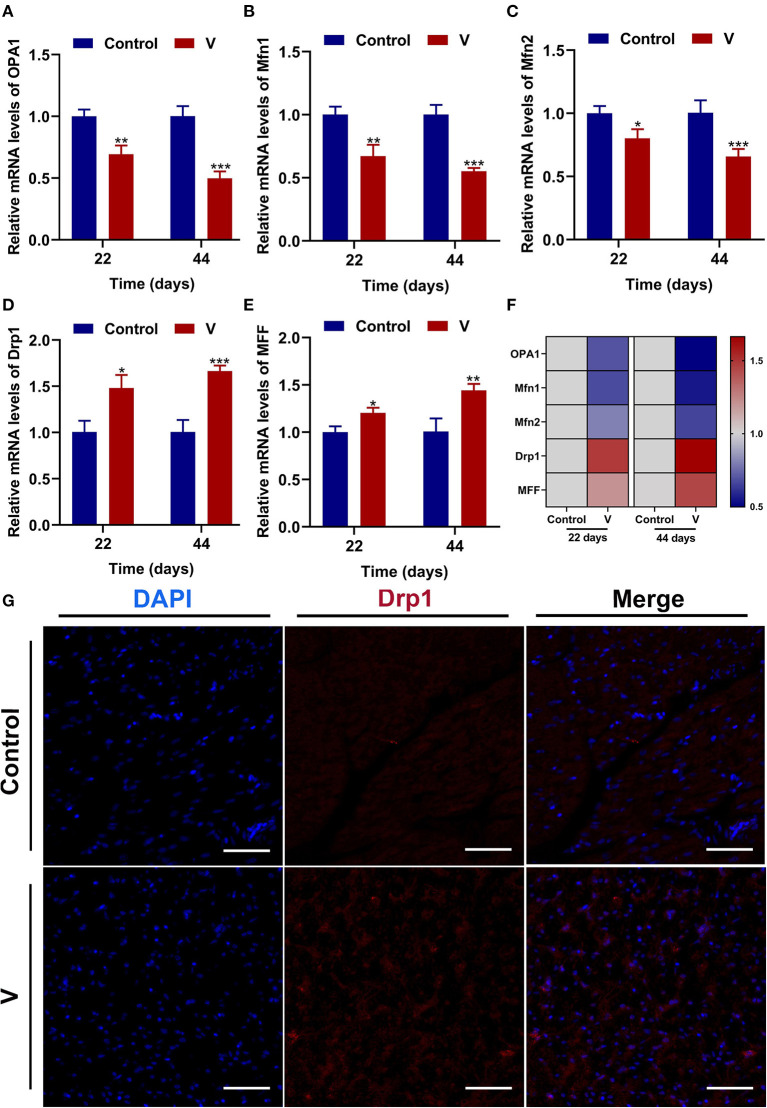
The effects of V on mitochondrial fission and fusion in the heart of ducks. **(A–E)** OPA1, Mfn1, Mfn2, Drp1, and MFF mRNA expression levels. **(F)** Heatmap analysis of mitochondrial fission and fusion-related mRNA levels. **(G)** Fluorescence microscope analysis of mitochondrial fission. In this picture, the nucleus straining is shown in blue, Drp1 straining is shown in red, and the bright red light is the fluorescent spot (×400 total magnification). Scale bar: 20 μm. *p < 0.05, **p < 0.01, and ***p < 0.001.

The mRNA levels of OPA1, Mfn1, and Mfn2 were markedly (*p* < 0.05, *p* < 0.01, or *p* < 0.001) lower in the V group than the control group on 22 and 44 days (Figures 4A–C). However, the mRNA levels of Drp1 and MFF were decreased remarkably (*p* < 0.05, *p* < 0.01, or *p* < 0.001) in the V group compared with the control group on 22 and 44 days (Figures 4D,E). The results of heatmap analysis was consistent with the variation of mRNA levels (Figure 4F). Meanwhile, on days 22 and 44, excessive V significantly (*p* < 0.001) elevated the protein levels of Mfn1 and Mfn2 (**Figures 6A,C,D**), but markedly (*p* < 0.01) descended the protein level of MFF (**Figures 6A,E**). In addition, V exposure enhanced the fluorescent localization signal of Drp1 in the myocardial cells on day 44 (Figure 4G).

### The Effects of V on Mitophagy in the Heart of Ducks

**Figure 5:**
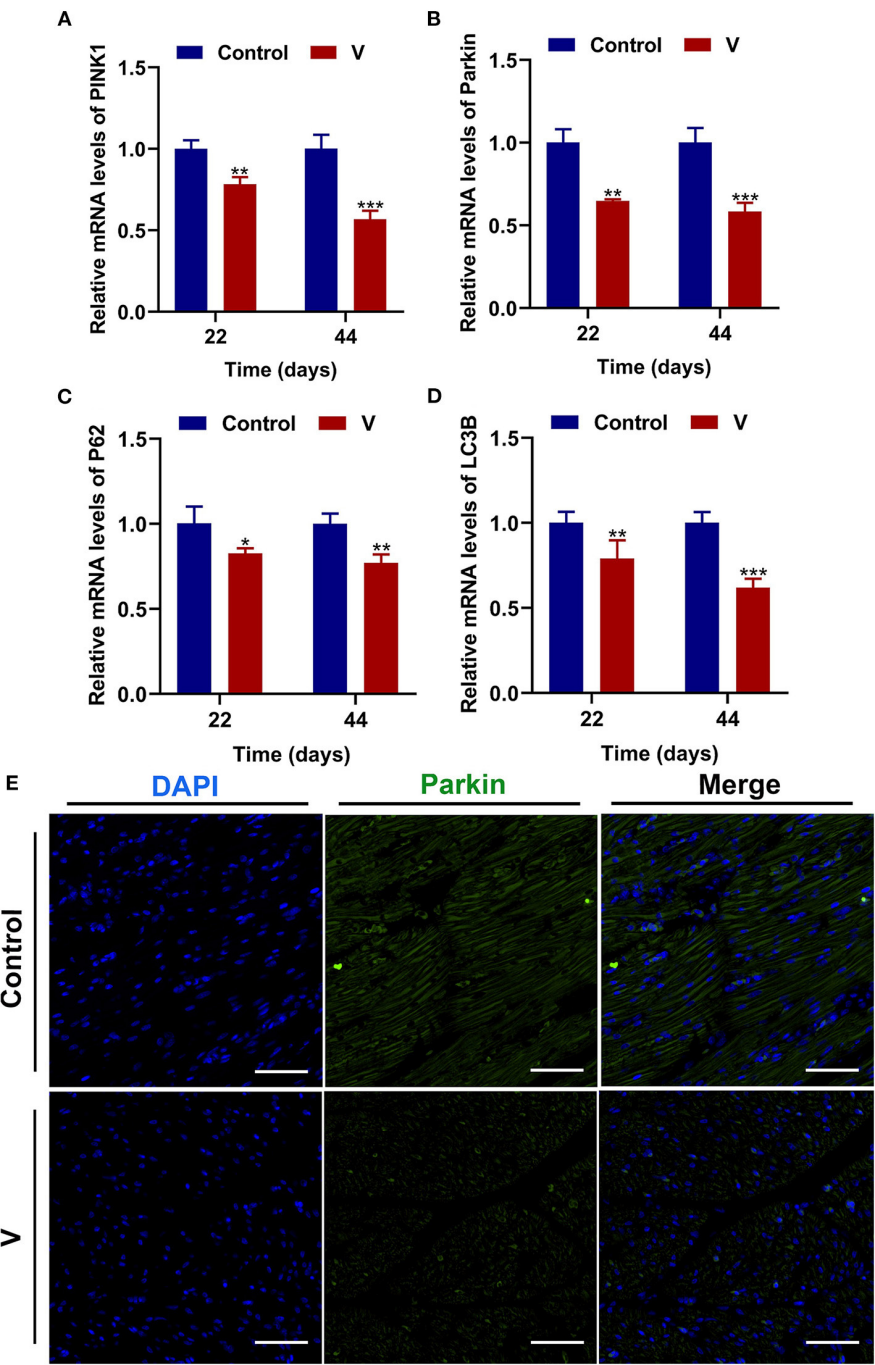
V exposure caused the inhibition of mitophagy in the heart of ducks. **(A–D)** PINK1, Parkin, P62, and LC3B mRNA levels. **(E)** Fluorescence microscope analysis of mitophagy. In this image, the nucleus straining is shown in blue, Parkin straining is shown in green, the bright green light is the fluorescent spot (×400 total magnification). The scale bar is 20 μm. *p < 0.05, **p < 0.01, and ***p < 0.001.

**Figure 6:**
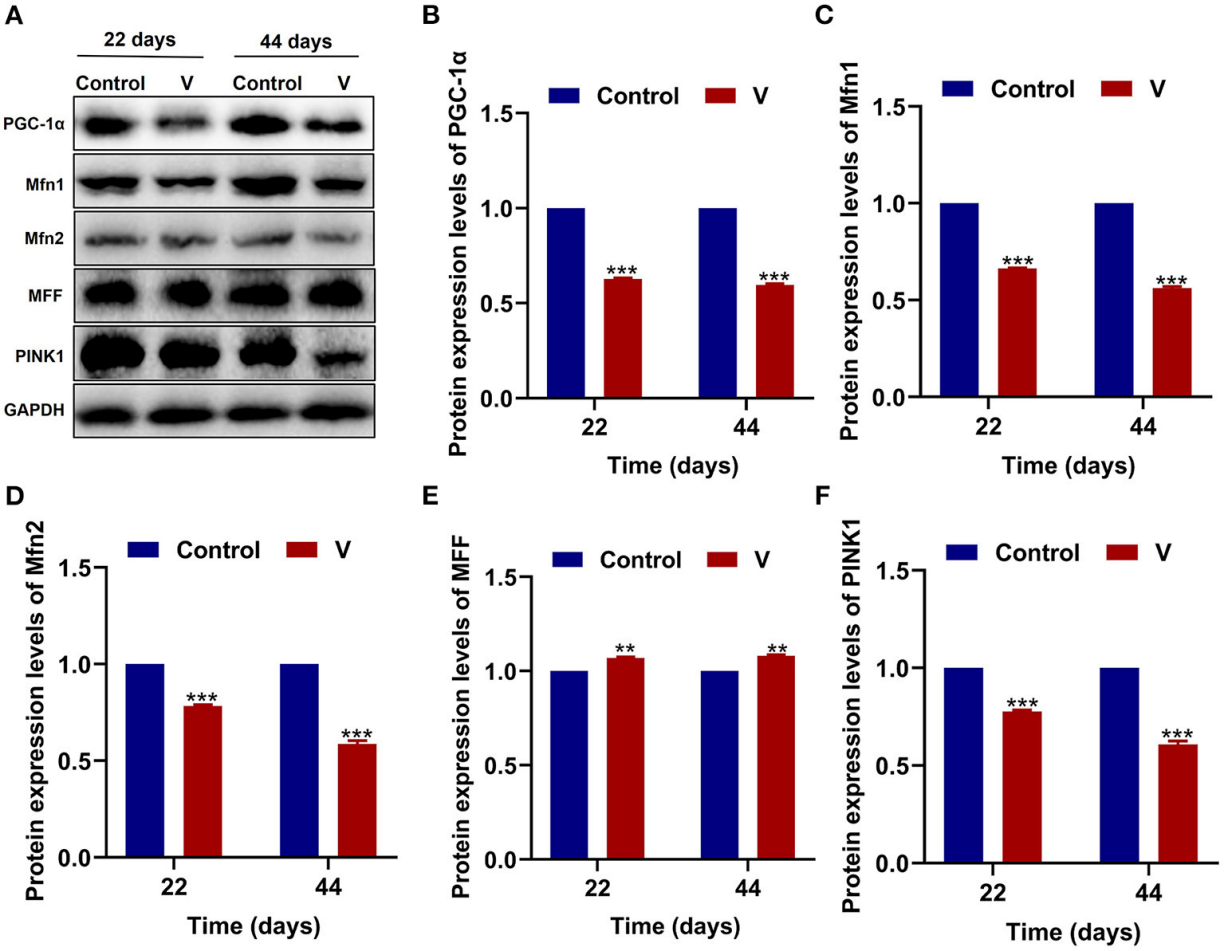
The effects of V on MQC-related proteins in the heart of ducks. **(A)** Protein band graph. **(B–F)** Protein levels of PGC-1α, Mfn1, Mfn2, MFF, and PINK1. **p < 0.01 and ***p < 0.001.

The mRNA levels of PINK1, Parkin, P62, and LC3B were reduced conspicuously (*p* < 0.05, *p* < 0.01, or *p* < 0.001) in the V group compared with the control group on 22 and 44 days (Figures 5A–D), and protein level of PINK1 was markedly (*p* < 0.001) lower in the V group than the control group on days 22 and 44 (Figures 6A,F). As shown in Figure 5E, the numbers of fluorescent spots of Parkin in the V group were decreased in comparison with the control group on 44 days.

## Discussion

V is an essential substance to support biological activities, but it also can cause organ injury when its concentration exceeds the body's tolerance. The heart with lots of mitochondria is one of V toxicity's main target organs, and mitochondria in which could be damaged by V toxicity leading to heart disorders. Previous studies have shown that V induced the impairment of mitochondrial electron transport by interacting with cysteine thiol, which in turn led to oxidative stress and mitochondrial dysfunction (32, 33). Simultaneously, MQC, as an important way to maintain mitochondrial function, can also be destroyed by the toxicity of heavy metal and its obstacle is usually accompanied by the occurrence of oxidative stress (34). Therefore, we speculate that V toxicity also causes damage to the MQC system, but the specific regulation mechanism of excessive V on oxidative stress and MQC deserves further exploration. Herein, this study was to explore the main events of V-induced cardiotoxicity via observing the variations of the antioxidant capacity and MQC-related factors in the heart of ducks.

V plays a key part in the growth and development of the body, but V exposure can cause functional and organic damage to many organs or systems. Previous studies have shown that V exposure could cause vacuolar degeneration and granular degeneration in hepatocytes, and it could also cause pathological changes in brain and kidney tissues (35, 36). In the present study, the sections of cardiac tissue from the V-treated group showed vacuolar and granular degeneration in cardiomyocytes. In addition, the results of the ultrastructure observed by the electron microscope showed that excessive V could trigger mitochondrial swelling and vacuolization in cardiomyocytes. These results strongly demonstrated that excessive V intake could induce cell damage in duck hearts.

V, a transition metal, participates in redox behavior and induces the production of ROS. Many scholars have pointed out that V induced cells to produce excessive amounts of ROS, leading to oxidative stress, which in turn triggered mitochondrial dysfunction (37, 38). Meanwhile, the antioxidative system can scavenge ROS through free radical scavenging antioxidant enzymes and non-enzymatic antioxidants (39). Notably, the antioxidant enzymes (SOD and CAT) play central roles in the antioxidant processes (40). Among them, ${\text{O}}_{2}^{•-}$ converts to H_2_O_2_ via the catalysis of SOD, then CAT catalyzes H_2_O_2_ into H_2_O and oxygen (41). MDA, the product of lipid peroxidation, the content of which reflects the injury degree of the oxidative damage. Thus, these antioxidant indexes are usually used as the basis to judge the degree of oxidative damage. In our study, excessive V elevated the levels of H_2_O_2_, MDA, and CAT, but increased T-SOD activity at 22 days and decreased at 44 days. According to the previous study, the antioxidant enzyme activities were enhanced but decreased after a while when the body suffered different degrees of oxidative damage (42, 43). Therefore, the changes of T-SOD activity might be related to the oxidative damage's intensity and time, which is higher at slight or transitory damage, opposite lower. Simultaneously, we speculated that V at a concentration of 30 mg/kg might cause severe damage to the heart of ducks, but the specific mechanism of the variations of T-SOD level deserves further study. Accordingly, these results indicated that excessive V could induce oxidative stress in the heart of ducks.

Oxidative stress is often accompanied by mitochondrial damage and then leads to mitochondrial dysfunction. Picca et al.'s study demonstrated that mitochondrial damage was induced by oxidative stress, MQC disorder, and the transfer of mitochondrial constituents (e.g., mitochondrial DNA, mtDNA) (44). However, MQC is a key mechanism for maintaining the mitochondrial function, and its regulatory mechanism under V exposure is unclear. Therefore, we investigated the regulatory mechanism of MQC under V exposure in duck hearts by measuring the levels of MQC-related mRNA and protein and their location in cardiomyocytes (45). PGC-1α, NRF-1, and TFAM are the key regulators of mitochondrial biogenesis (46). TFAM is activated by the combination of PGC-1α and NRF-1 to regulate the mitochondrial biogenesis transcription process for mitochondrial homeostasis. Herein, high dietary V decreased the mRNA levels of PGC-1α, NRF-1, and TFAM, as well as the protein level of PGC-1α in the heart of ducks, suggesting that excessive V could induce the impairment of mitochondrial biogenesis in the heart of ducks. In addition, mitochondrial fission and fusion are regarded as critical processes to determine the mitochondrial shape (47). Drp1 is recruited to the mitochondrial outer membrane and interacts with multiple receptor proteins like mitochondrial fission factor (MFF) to achieve the segregation of damaged mitochondria (48, 49). However, mitochondrial fusion is a mechanism that mediates OPA1, Mfn1, and Mfn2 to form OPA1 and Mfn1/2 protein complexes to achieve stable mitochondrial membrane fusion (50). In this paper, excessive V upregulated the mRNA and protein levels of Drp1 and MFF in the heart of ducks, and downregulated the mRNA and protein levels of OPA1, Mfn1, and Mfn2, which indicated that V promoted mitochondrial fission in the heart of ducks but inhibited mitochondrial fusion. In addition, there are mainly PINK1/Parkin, Bnip3/Nix, and FUNDC1 pathways to regulate mitophagy, and PINK1/Parkin pathway is closely related to the regulation of mitochondrial function (51, 52). In this pathway, PINK1 selectively acts on the upstream of Parkin when mitochondria depolarize and accumulate on the damaged mitochondria to activate Parkin's translocation to the mitochondria, which then triggers mitophagy (53). Meanwhile, LC3B binds to P62 accumulated in the ubiquitinated mitochondrial matrix to initiate mitochondrial autophagy by mediating the entry of ubiquitinated substrates into autophagosomes (52). In our study, excessive V could reduce the mRNA levels of Parkin, PINK1, P62, LC3B, and the protein level of PINK1 leading to defective mitophagy in cardiomyocytes. Yu et al.'s (54) research showed that the high dose of heavy metal could cause serious damage to cells and mitochondria by inhibiting the mitophagy pathway. Therefore, we speculated that excessive V could damage cardiomyocytes severely, and mediated the defects of mitophagy by inhibiting the PINK1/Parkin signaling pathway, but the effects on other mitophagy pathways need to be further studied. Collectively, we found that V could induce MQC disorder in the heart of ducks. However, the interrelationship of multiple mechanisms within MQC needs further study.

Taken together, we found that excessive V could induce oxidative stress and MQC disorder in the heart of ducks, which provides a basis for exploring the toxicological mechanisms of V in the heart.

## Data Availability Statement

The original contributions presented in the study are included in the article/supplementary material, further inquiries can be directed to the corresponding authors.

## Ethics Statement

The animal study was reviewed and approved by the Animal Ethics Committee of Jiangxi Agricultural University (Approval ID: JXAULL-2020-32).

## Author Contributions

ZX, CX, TX, GL, YY, GH, and HC were responsibility for experiment conception, design, and practice. ZX, CX, and TX were involved in the drafting of the article. HC, FY, CZ, and XG revised the article.

## Funding

This work was supported by the National Natural Science Foundation of China (No. 31902333) and the Science and Technology Plan of Education Department of Jiangxi Province (GJJ190216).

## Conflict of Interest

The authors declare that the research was conducted in the absence of any commercial or financial relationships that could be construed as a potential conflict of interest.

## Publisher's Note

All claims expressed in this article are solely those of the authors and do not necessarily represent those of their affiliated organizations, or those of the publisher, the editors and the reviewers. Any product that may be evaluated in this article, or claim that may be made by its manufacturer, is not guaranteed or endorsed by the publisher.
